# Editorial: EEG and fMRI for Sleep and Sleep Disorders–Mechanisms and Clinical Implications

**DOI:** 10.3389/fneur.2021.749620

**Published:** 2021-09-28

**Authors:** Xi-Jian Dai, Jihui Zhang, Yongjun Wang, Yan Ma, Kuangyu Shi

**Affiliations:** ^1^Shenzhen Mental Health Centre, Shenzhen Kangning Hospital, Shenzhen, China; ^2^Guangdong Mental Health Center, Guangdong Provincial People's Hospital, Guangdong Academy of Medical Sciences, Guangzhou, China; ^3^Osher Center for Integrative Medicine, Division of Preventive Medicine, Brigham and Women's Hospital, Harvard Medical School, Boston, MA, United States; ^4^Department of Nuclear Medicine, University of Bern, Bern, Switzerland

**Keywords:** EEG, functional magnetic resonance imaging, sleep disorders, sleep deprivation, obstructive sleep apnea, shift work, sleepiness, restless legs syndrome

## Introduction

In modern society, more and more people undergo an increased incidence of sleep disorders. Sleep disorders are a hallmark of most neurological diseases, yet sleep disorders themselves are commonly found to have far more health impact than previously thought. While it is generally accepted that sleep is critical for normal brain function, there is as yet no agreement upon the function of sleep. Although there has been a surprising upsurge in neuroimaging findings that address the brain's structural and functional changes associated with sleep disorders, sleep is less frequently studied using imaging techniques than many neuropsychiatric disorders (1). Thus, modern sleep medicine requires a multi-modal approach to investigate brain changes occurring in response to or as causative mechanisms of sleep-related neuropathology.

Electroencephalography (EEG) and functional magnetic resonance imaging (fMRI) are key neuroimaging techniques used to explore the mechanisms of sleep disorders. Sleep spindle, K-complex, and sleep slow wave are specific sleep electrical waves, which are very important for us to understand the mechanisms and clinical implications of sleep. Clinically, human sleep and sleep pathology are defined based upon scalp recorded electrical potentials (EEG), which have provided seminal information on the changes of brain electrophysiology that are associated with sleep pathologies and pathologies that adversely affect sleep. In this issue, EEG and fMRI have been utilized to examine changes associated with pathologies of sleep. The aim of this Research Topic is to contribute to a better understanding of the etiology of sleep disorders.

## Sleep Deprivation

This specific issue includes two studies focusing on sleep deprivation (SD). In one study by Zeng et al., the authors reported regional brain activity alteration in healthy subjects after a total of 36 h of SD relative to after normal sleep, using a percent amplitude of fluctuation (PerAF) method. They found that SD resulted in a 2.23% decrease in accuracy rate and an 8.82% increase in reaction time, and the SD was associated with decreased PerAF in the bilateral dorsolateral prefrontal cortex, which positively correlated with the accuracy rate.

In the second study by Li et al., these authors examined the association of the thalamus with SD-related emotion changes. In their study, positive emotions and psychomotor performance were reduced, while negative emotions were increased after a total of 36 h of SD. The left thalamus was selected as a region of interest for seed point functional connectivity, and decreased functional connectivity was found between this area and left middle temporal gyrus, left inferior frontal gyrus, right thalamus, right inferior temporal gyrus, left middle temporal pole gyrus, right calcarine, left cuneus, left rectus, and left medial superior frontal gyrus. Among these connectivity pairs, the decreased functional connectivity between the left thalamus and the left inferior frontal gyrus negatively correlated with negative emotion changes.

## Obstructive Sleep Apnea

Two studies focusing on obstructive sleep apnea were included in our specific issue. The first study by Zhou et al. examined functional connectivity changes in the bilateral hippocampus in treatment-naïve patients with moderate or severe obstructive sleep apnea. It was found that extensive abnormal functional connectivity of the right hippocampus may contribute to the joint effect of intermittent nocturnal hypoxia and sleep fragmentation, while the reduced functional connectivity between the left hippocampus and the anterior cerebellar lobe was more likely caused by a disturbed sleep architecture.

The second study by Wu Y. et al. summarizes the literature on resting-state EEG and resting-state fMRI (rs-fMRI) studies in patients with obstructive sleep apnea. Evidence from EEG-related studies showed that patients with obstructive sleep apnea were associated with increased power of δ and θ in the frontal and central regions, and their slow-wave activity showed a positive correlation with their apnea-hypopnea index. For rs-fMRI studies, the prefrontal cortex and insula may be the vital regions of obstructive sleep apnea, and these areas strongly correlated with the severity of diseases. Furthermore, some large-scale brain networks, such as the default-mode network, salience network, and central executive network, play important roles in the pathology of obstructive sleep apnea. Finally, they proposed four suggestions about the intervention therapy for obstructive sleep apnea.

## Narcolepsy/Sleepiness

In this specific issue, three studies focusing on narcolepsy/sleepiness-related issues were included. One study by Bioulac et al. found drug-free sleepy patients with attention deficit hyperactivity disorder (ADHD) had significantly shorter sleep latency relative to normal controls during the maintenance of wakefulness test, and the difficulty to remain awake during soporific circumstances observed in these patients with ADHD cannot be explained by changes in the kinetic of sleep pressure buildup.

The second study by Deng et al. investigated the dynamic cerebral autoregulation, as assessed by phase difference using transfer function analysis, in patients with central disorders of hypersomnolence. The study found that these patients were associated with impaired dynamic cerebral autoregulation and that recovery is possible after treatment in patients with narcolepsy type 1.

The third study by Zhu et al. applied simultaneous EEG-fMRI to examine the brain network topological characteristics in patients with narcolepsy type 1 (NT1) disorder. The study found lower global brain network efficiency during the N2 sleep stage and reduced small-world attributes in NT1 patients, relative to normal controls.

## Shift Work

Two studies examined brain network changes caused by shift work. One study by Ning et al. first identified seven brain networks and evaluated their granger causality connections. The study reported increased inflows from the anterior default mode network (aDMN) to sensorimotor network (SMN) and left frontoparietal network (LFPN) to salience network (SN), and negative associations with attention and visuospatial scores. In another study by Wu X. et al., it was found that night shift work could lead to poor sleep quality and lower melatonin levels, as well as a series of changes in regional brain activity and connectivity.

## Restless Legs Syndrome

One study by Sun et al. evaluated the sleep characteristics and their related risk factors among Parkinson's disease (PD) patients with and without restless legs syndrome (RLS). This study provided evidence that PD patients with RLS exhibited worse nocturnal sleep than PD patients without RLS. They also observed several risk factors, such as periodic limb movements during sleep in PD patients with RLS.

## Sleep in Patients With PTSD

One study by Laxminarayan et al. compared spectral power and synchrony features in subjects with post-traumatic stress disorder (PTSD) during two consecutive nights of sleep. They developed a classifier that could be used to discriminate subjects with and without PTSD using robust stage-independent and whole-night features from EEG signals.

## Conclusions

Together, this issue features articles that address the relationships between sleep disorders and changes in brain electrophysiology, structure, and function using EEG and neuroimaging methods. We hope this issue will provide seminal information for a better understanding of how sleep disorders affect our brain and behavior. We believe that this issue will stimulate discussions beyond those working in the field, in particular in those patients suffering from sleep disorders. Public health guidance should pay more attention to sleep problems and advocate healthy lifestyle habits.

## Author Contributions

All authors listed have made a substantial, direct and intellectual contribution to the work, and approved it for publication.

## Conflict of Interest

The authors declare that the research was conducted in the absence of any commercial or financial relationships that could be construed as a potential conflict of interest.

## Publisher's Note

All claims expressed in this article are solely those of the authors and do not necessarily represent those of their affiliated organizations, or those of the publisher, the editors and the reviewers. Any product that may be evaluated in this article, or claim that may be made by its manufacturer, is not guaranteed or endorsed by the publisher.
